# Retraction Note to: LncRNA HCG11 regulates proliferation and apoptosis of vascular smooth muscle cell through targeting miR‑144‑3p/FOXF1 axis in atherosclerosis

**DOI:** 10.1186/s40659-021-00352-4

**Published:** 2021-09-09

**Authors:** Yi Liu, Xiyun Cui, Cong Wang, Sihai Zhao

**Affiliations:** 1 Department of Clinical Laboratory, The First Affiliated Hospital of Xi’an Jiaotong University, Xi’an, 710061 Shaanxi China; 2 Department of Clinical Laboratory, Weapon Industry 206 Hospital, Xi’an, 710061 Shaanxi China; 3 Laboratory Animal Center, Xi’an Jiaotong University School of Medicine, Xi’an , 710061 Shaanxi China

## Retraction to: Biol Res (2020) 53:44 https://doi.org/10.1186/s40659-020-00306-2

The authors have retracted this article because following verification, the results of the statistical analysis of the data presented in Fig. 4D and Fig. 6D were identified to be incorrect; specifically, the TUNEL assay results indicated that microRNA-144 could not reverse the effects of HCG11 on apoptosis, which affected the overall conclusions of the study.

All authors agree to this retraction.

